# Malaria during pregnancy and transplacental transfer of Kaposi sarcoma-associated herpesvirus (KSHV) antibodies: a cohort study of Kenyan mother and child pairs

**DOI:** 10.1186/s13027-020-00336-1

**Published:** 2020-11-26

**Authors:** Katherine R. Sabourin, Sidney Ogolla, Ibrahim I. Daud, Conner L. Jackson, Wendell Miley, Nazzarena Labo, Denise Whitby, Rosemary Rochford

**Affiliations:** 1 Department of Epidemiology, University of Colorado Anschutz Medical Campus, Aurora, USA; 2 Department of Immunology and Microbiology, CU School of Medicine, University of Colorado Anschutz Medical Campus, 12800 E. 19th Ave, RC1N P18-9403D, Aurora, CO 80045 USA; 3 Centre for Global Health Research, Kenya Medical Research Institute, Kisumu, Kenya; 4 United States Army Medical Research Laboratories, Kericho, Kenya; 5 Department of Biostatistics and Informatics, University of Colorado Anschutz Medical Campus, Aurora, USA; 6 Frederick National Laboratory for Cancer Research, Leidos Biomedical Research, Frederick, MD USA

**Keywords:** KSHV, Transplacental transfer, Malaria, Africa, Mother-child pairs, Antibodies

## Abstract

**Background:**

Kaposi sarcoma-associated herpesvirus (KSHV) seroprevalence in sub-Saharan African children can range up to 50% by age 2 years but factors affecting early age of KSHV infection are not well understood. Malaria during pregnancy has been associated with hindered transplacental transfer of antibodies to several pathogens but whether it affects transplacental transfer of KSHV antibodies is unknown. We aimed to determine if *in utero* malaria exposure reduced the transfer of KSHV antibodies across the placenta.

**Methods:**

A cohort study in Kisumu, Kenya enrolled pregnant women at their first antenatal clinic (ANC) visit and followed them through delivery. We included 70 KSHV-positive, HIV-negative mothers and their children. KSHV antibody levels were measured by ELISA (K8.1, ORF73) and multiplex assay (K8.1, ORF73, K10.5, ORF38, ORF50). Transplacental transfer of antibodies was measured by the cord to maternal blood ratio (CMR) of KSHV antibodies. Malaria during pregnancy was defined as detection of *Plasmodium falciparum* (*Pf*) DNA at any ANC visit or delivery. Among women with malaria during pregnancy, we examined time of last malaria infection prior to delivery (< 27 vs. 27+ weeks gestation) and malaria incidence rate (MIR) (episodes/100 person-weeks).

**Results:**

KSHV seroprevalence (positive for K8.1 or ORF73 by ELISA) among pregnant women was 88%. Neither malaria during pregnancy, malaria infection timing, nor MIR were associated with maternal delivery KSHV antibody blood levels. Maternal delivery and cord blood KSHV antibody levels were highly correlated but these correlations did not differ by malaria during pregnancy. KSHV transplacental antibody transfer was not associated with malaria during pregnancy, malaria infection timing, nor MIR.

**Conclusions:**

Malaria during pregnancy does not appear to affect transfer of KSHV antibodies across the placenta.

**Supplementary Information:**

The online version contains supplementary material available at 10.1186/s13027-020-00336-1.

## Introduction

Kaposi sarcoma-associated herpesvirus (KSHV) is the etiological agent for Kaposi sarcoma (KS), one of the ten most common cancers in Kenya [1]. In sub-Saharan Africa where KSHV is endemic, primary infection predominately occurs in young children. A study of Ugandan children found that 31% were seropositive by five years of age [2] and in Zambia up to 50% of children were found to be seropositive by age two years [3].

Maternally derived immunoglobulin G (IgG) antibodies transported across the placenta provide passive humoral immunity that protects infants for the first few months of their lives. The levels of antibodies transferred from mother to fetus determine the duration and strength of protection against infection. Placental malaria has been associated with reduced transfer of total IgG antibodies [4, 5] and antibodies against tetanus [4, 6], herpes simplex virus 1 (HSV-1), varicella-zoster virus (VZV), respiratory syncytial virus (RSV) [5], *S. pneumonia* and measles [7, 8] and malaria during pregnancy has been associated with reduced transfer of antibodies for Epstein-Barr virus (EBV) [9]. Altered transfer of pathogen specific antibodies may result in the infant being susceptible to infections early in life.

Although malaria has been associated with reduced transplacental transfer of antibodies to other pathogens, the effect of malaria infection during pregnancy on maternal transfer of KSHV antibodies is relatively unknown. We used samples from the Chulaimbo Antenatal Postnatal (CHAP) cohort of pregnant women and their newborns enrolled from Kisumu District in Kenya, a region with a high KSHV seroprevalence that is also malaria holoendemic, to determine if malaria infection during pregnancy reduces the transplacental transfer of KSHV antibodies from mother to child.

## Methods

### Study population

The CHAP study was a longitudinal cohort of pregnant women and their children initiated in 2011. The original study was designed to explore factors that determine early age of infection with EBV. The study population is described elsewhere in more detail [10, 11]. In summary, women of all gravidities were enrolled at the Chulaimbo County Hospital, Chulaimbo, Kenya, which serves a predominately rural population, during their first antenatal clinic (ANC) visit between June and November 2011. Women were enrolled if they resided within 10 km of the hospital, were willing to return for follow-up, had a singleton pregnancy, vaginal delivery, no blood transfusion ≤24 h before delivery, nor had other complications or clinical illness besides malaria during pregnancy (< 1% of women). Women were followed-up at each ANC visit (up to four total) and at delivery, if they delivered at the study hospital.

Informed consent was obtained from each study participant. Research was performed in accordance with the Declaration of Helsinki and all protocol and consent forms were approved by the Scientific and Ethical Review Unit (SERU) at the Kenya Medical Research Institute (KEMRI), the Colorado Multiple Institutional Review Board (COMIRB), and the State University of New York (SUNY) Upstate Medical University (where study was initiated).

### Data collection

#### Demographics

At enrollment, information on demographics and pregnancy history was collected including data on the woman’s tribe, education, marital status, age, gravidity, and bed net use at time of enrollment.

#### Blood collection and processing

Pregnant women provided finger stick blood samples at each ANC visit and 2-4 mL of venous blood at enrollment and within 12 h of delivery. An incision on the maternal side of the placenta was made to collect an intervillous blood (IVB) sample [12]. Cord blood was collected from the umbilical vein immediately after delivery, as previously described [9]. For venous blood draws, plasma was removed and saved following separation of peripheral blood mononuclear cells (PBMCs) over Ficol-Hypaque. All samples were stored at − 80 °C until further analysis.

#### Determination of malaria status

Maternal, placental, and cord malaria status were determined by detection of *Plasmodium falciparum* (*Pf*) DNA as described previously [10]. A woman was defined as having a malaria infection during pregnancy if *Pf* DNA was detected by quantitative polymerase chain reaction (qPCR) and/or blood smear [12]. Subsequently, an infant was considered exposed to malaria if the mother had malaria during pregnancy.

#### Prenatal infection status

Presence of antibodies to either KSHV open reading frame (ORF)73 or K8.1 in plasma taken at enrollment and tested by enzyme-linked immunosorbent assays (ELISA) was used to indicate whether pregnant women were KSHV seropositive [13]. All pregnant women were tested for human immunodeficiency virus (HIV) based on Kenyan Ministry of Health national guidelines and only HIV-negative women were analyzed for this study. At each ANC visit, pregnant women provided urine and stool samples which were tested for parasites. Women were considered to have had helminthiasis during pregnancy if they tested positive for hookworm, *Trichuris trichura*, *Ascaris lumbricodes*, *E. histolytica*, *Giardia lamblia,* stronglyoide*s,* or *Schistosoma hematobium.*

#### Newborn characteristics

Child sex, birthweight (grams), and gestational age were collected at birth. Gestational age was calculated as the number of weeks from the last reported menstrual period to delivery. Gestational ages over 45 weeks were truncated to 45 weeks as that was the longest reasonable gestational age in the sample (*n* = 2). Children were considered preterm if born < 38 weeks gestational age and low birth weight if weighing ≤2500 g at birth.

#### Determination of Hypergammaglobulinemia

Total IgG was measured in maternal venous blood at delivery using the Human Total IgG ELISA kit from eBioscience (San Diego, CA) following the manufacturer’s instructions. Hypergammaglobulinemia in pregnant women was defined as having levels of total IgG > 30 mg/ml as per kit instructions.

#### KSHV ELISA

IgG antibodies to K8.1 and ORF73 were tested using KSHV ELISA as previously described [13]. Plasma samples and controls were diluted 1:100 in assay buffer in both K8.1 and ORF73 coated ELISA plates in a final volume of 100ul. Cut-off values for each plate were calculated as a constant value of 0.346 (for K8.1) or 0.350 (for ORF73) plus the average background subtracted optical density (OD) of the plates’ negative controls to account for minor plate-to-plate variability. Constant values were chosen based on receiver operating characteristic (ROC) analyses of each assay using a panel comprising 86 blood donors from the USA and 88 patients diagnosed with KSHV-associated disease or KSHV DNA detected by qPCR. The best classifying and/or least misclassifying cut points (maximizing Youden’s index) were chosen.

#### KSHV serology by bead-based multiplex assay

The same samples were tested for antibodies to KSHV proteins K8.1, ORF73, K10.5, ORF38, and ORF50 using the bead-based multiplex assay as described [14]. Healthy North American adult blood donors considered at low risk of KSHV infection were used as negative assay controls. North American adult patients with active KSHV-associated disease or history of disease and detectable KSHV DNA were used as positive assay controls. The median fluorescence intensity (MFI) across all counted beads was computed for each sample and recorded after subtracting the background fluorescence.

### Analysis

#### Analytic sample

Of the 200 women enrolled in the original study we included 70 KSHV-positive, HIV-negative women who delivered at the study hospital and had complete maternal and cord blood sample pairs. Because we wanted to look at transplacental transfer of KSHV antibodies, we only included KSHV-positive women (Additional file 1).

#### Exposures

Malaria during pregnancy was defined as ever occurring if *Pf* was detected at any of the attended ANC visits or at delivery in either maternal or cord samples. To measure malaria burden during pregnancy and also account for differences in the gestational ages at which women were enrolled, we calculated a malaria incidence rate (MIR). MIR was calculated as the number of malaria episodes divided by the total weeks a pregnant woman was in the study before delivery multiplied by 100. Number of malaria episodes was determined by the number of times a pregnant woman was considered positive for *Pf* by qPCR. Consecutive positive detections by qPCR were considered a single malaria episode unless there was a two-month gap between malaria positive ANC visits. Pregnant women should have received Sulfadoxine-pyrimethamine (SP) at each ANC visit as a malaria prophylaxis and so we expected that any infection after two months would be a new exposure. Finally, we examined whether timing of the most recent malaria episode prior to delivery had any effect on maternal transplacental transfer of KSHV antibodies. Timing of malaria was defined as early most recent malaria exposure (< 27 weeks gestational age) vs. late most recent malaria exposure (27+ week gestational age).

#### Outcome

KSHV antibody responses were measured by ELISA (K8.1 and ORF73) and multiplex (K8.1, ORF73, K10.5, ORF38, and ORF50). We examined maternal venous blood KSHV antibody levels at delivery. Transplacental transfer of antibodies was measured as the cord to maternal blood ratio (CMR) for each protein.

#### Data analysis

Univariate descriptive statistics were calculated using Chi-squared or Fisher’s exact tests for categorical variables, and student's t-tests for continuous normally distributed variables. Mann Whitney U tests were used to model the relationship between malaria during pregnancy, or among women with malaria during pregnancy, timing of last malaria infection and maternal venous blood KSHV antibody levels at delivery. Spearman’s correlation coefficient was used for the analysis of MIR with maternal venous blood KSHV antibody levels at delivery, among women with a malaria infection during pregnancy. Pearson’s correlation coefficient was used to determine the correlations between KSHV antibody levels in maternal venous blood taken at delivery and neonatal cord blood. KSHV antibody levels measured by multiplex were log-transformed. Linear regression was used to model the associations between malaria during pregnancy, and among those with malaria during pregnancy, timing of last malaria infection and MIR with log-transformed KSHV antibody CMR. For adjusted analysis, maternal and child characteristics identified a priori to be associated with both malaria and transplacental transfer of KSHV antibodies were assessed as potential confounders. These included: maternal tribe, education, marital status, parity, bed net use during pregnancy, worm infection during pregnancy, and hypergammaglobulinemia, and child’s gestational age and birthweight. Associations between potential confounders and either exposure or KSHV antibody CMR were modeled using Mann Whitney U, Kruskal Wallis, Chi-squared, Fisher’s exact test, or Spearman correlation coefficient, as appropriate. Variables associated with both the exposure of interest and KSHV antibody CMR by *p*-value < 0.2 were selected as potential candidates and included in the final model if they changed the association estimate by ≥10%. For each exposure, *p*-values were adjusted for multiple comparisons using a false discovery rate (FDR) adjustment. All analyses were completed using SAS 9.4.

### Sensitivity analysis

To determine if our sample contained selection bias, we compared demographic and clinical characteristics of pregnant women enrolled in the study by whether they were included in the final analysis, excluded because they were HIV-positive, and excluded for any other reason. Chi-squared, Fisher’s exact test, and ANOVA were used, as appropriate, to compare univariate statistics across the three groups.

## Results

### Study population characteristics

Of the 200 women enrolled in the study with KSHV ELISA results at baseline (*n* = 199), 175 (88%) were KSHV seropositive. Pregnant women tested positive for KSHV by detection of ORF73 alone (*n* = 36, 18%), K8.1 alone (*n* = 8, 4%) or either K8.1 or ORF73 (*n* = 131, 66%). Our final analysis included 70 (35%) KSHV-positive, HIV-negative women who delivered at the study hospital with complete maternal venous blood and cord blood sample pairs (Additional file 1).

Pregnant women were an average of 22 years old at enrollment, primarily of Luo tribe (87%), married (67%), and with upper primary schooling or higher (79%). Helminths were detected during pregnancy in 23% of women and hypergammaglobulinemia in 27% of women. Almost all the newborns were of normal birthweight (91%) and most were full term (71%) (Table 1).

**Table 1:** Maternal demographics, clinical characteristics, and pregnancy history and newborn clinical characteristics (*N* = 70)

		Malaria during pregnancy		
	Total (***N*** = 70)	Never (***n*** = 32)	Any (***n*** = 38)	***p***-value
**Maternal characteristics**				
**Mother’s age at enrollment (mean) [sd]**	22 [6.2]	22.3 [6.5]	21.7 [6]	0.69
**Mother’s tribe:** Luo vs. Luhya	61 (87.1)	28 (87.5)	33 (86.8)	1.00
**Mother's education**: Upper primary school or higher vs. Lower primary school or lower	55 (78.6)	25 (78.1)	30 (78.9)	0.93
**Mother's marital status**: Married vs Single/widowed	47 (67.1)	21 (65.6)	26 (68.4)	0.80
**Maternal bed net use**: Yes vs. No	68 (97.1)	30 (93.8)	38 (100)	0.21
**Maternal gravidity**				
Nulliparous	25 (35.7)	9 (28.1)	16 (42.1)	0.27
Primiparous	22 (31.4)	13 (40.6)	9 (23.7)	
Multiparous	23 (32.9)	10 (31.3)	13 (34.2)	
Worm infection during pregnancy**	16 (22.9)	5 (15.6)	11 (28.9)	0.28
**Hypergammaglobulinemia (Adjusted venous blood concentration):** > 30 mg/ml	19 (27.1)	6 (18.8)	13 (34.2)	0.14
**Sulfadoxine-pyrimethamine (SP) given during pregnancy or delivery**	70 (100)	32 (100)	38 (100)	1.00
**Total antenatal clinic (ANC) visits**				
1	4 (5.7)	3 (9.4)	1 (2.6)	0.008*
2	6 (8.6)	4 (12.5)	2 (5.3)	
3	17 (24.3)	12 (37.5)	5 (13.2)	
4	43 (61.4)	13 (40.6)	30 (78.9)	
**Neonate characteristics**				
**Child sex**: Male vs Female	35 (50)	20 (62.5)	15 (39.5)	0.05
**Child birthweight (mean) [sd]**	3176 [415]	3169 [396]	3182 [436]	0.90
Normal (> 2500 g) vs low birthweight (≤2500 g)	64 (91.4)	30 (93.8)	34 (89.5)	0.68
**Gestational age at birth (mean) [sd]**	38.8 [3.4]	38.4 [3.6]	39.2 [3.1]	0.38
Full term (≥38 weeks) vs Preterm (< 38 weeks)	50 (71.4)	22 (68.8)	28 (73.7)	0.65
**Among women with malaria during pregnancy (*n*** **= 38)**				
**Placental malaria**	–	–	4 (10.5)	< 0.001*
**Gestational age at latest malaria exposure: >** 27+ weeks vs ≤26 weeks			24 (63.2)	0.10
**Malaria incidence rate (events/100 person-weeks) (mean) [sd]**	–	–	8.8 [5.2]	< 0.001*

Data are presented as number (percent) unless otherwise specified

*Abbreviation*: *sd* standard deviation

*p-values < 0.05 considered statistically significant

**All variables have complete data except worm infection which was missing for 5(15.6%) women with no malaria during pregnancy and 2(5.3%) with malaria during pregnancy

### Malaria infection during pregnancy

All women who were positive for malaria by blood smear were also positive by qPCR. All but two women reported using a bed net during their pregnancy and all enrollees received SP at least once during pregnancy or delivery. Among pregnant women with malaria infection, only four (11%) had placental malaria and the average MIR was 8.8 per 100 person-weeks. The overall MIR of our study participants (both with and without malaria during pregnancy) was 4.7 malaria episodes per 100 person-weeks. The most recent malaria infection prior to delivery was during the last trimester (27+ weeks) for more than half of pregnant women who had any malaria infection during pregnancy (*n* = 24, 63%) (Table 1).

There were no significant differences in maternal or child demographics or characteristics for women with malaria infection during pregnancy compared to women with no malaria infection except that children born to mothers with malaria during pregnancy were more likely to be female (61% vs. 38%; malaria exposed vs. unexposed, respectively) and mothers with malaria infection were more likely to come for all four ANC visits. (79% vs. 41%; malaria exposed vs. unexposed, respectively) (Table 1).

### Maternal KSHV antibody levels at delivery

Maternal levels of pathogen specific antibodies have been reported to influence the levels in their neonates. We first looked at levels of maternal venous blood KSHV antibodies by whether women had a malaria infection during pregnancy. Maternal KSHV antibody levels appeared to be higher in women who had a malaria infection during pregnancy for all antigens except K10.5 as measured by multiplex, though differences in levels were not significant. Among women with a malaria infection, there were no significant differences between maternal KSHV antibody levels at delivery and timing of the most recent malaria infection prior to delivery, and there was no significant correlation between maternal KSHV antibody levels at delivery and MIR (Additional file 2).

**Fig. 1:**
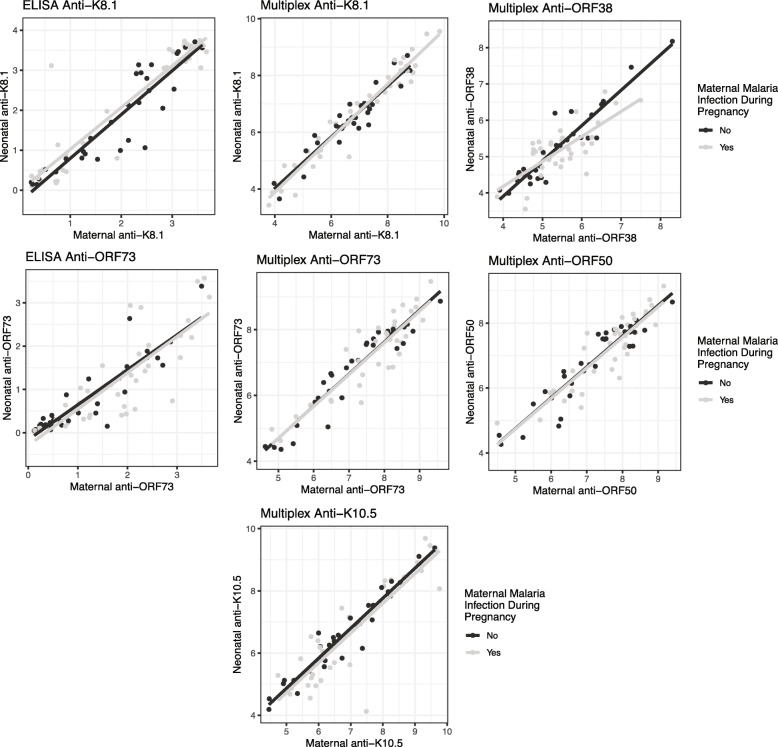
Correlations between KSHV antibody levels in maternal delivery and in cord blood by malaria during pregnancy status. Footnote: Pearson’s correlation coefficients for KSHV antibody levels in maternal venous blood taken at delivery (x-axis) and KSHV antibody levels in neonatal cord blood (y-axis) stratified by maternal malaria infection during pregnancy (ever/never) status. Antibody levels measured by multiplex were log transformed. *P*-values< 0.05 were considered statistically significant

We also compared KSHV antibody levels in maternal venous blood at delivery to levels in neonate cord blood. KSHV antibody levels in neonatal cord and maternal venous blood taken at delivery were highly correlated for all anti-KSHV antibodies but these correlations did not differ by whether women had a malaria infection during pregnancy (Fig. 1).

### Transplacental transfer of KSHV antibodies

We next determined if malaria infection during pregnancy interferes with the transplacental transfer of antibodies against KSHV. We found no statistically significant differences in the transplacental transfer of KSHV antibodies between mothers with and without malaria infection during pregnancy. Among women with malaria infection during pregnancy, there was no statistically significant difference in the transplacental transfer of KSHV antibodies by whether the most recent malaria infection prior to birth occurred before or after 26 weeks gestational age. There was also no statistically significant linear association between MIR and KSHV antibody transplacental transfer levels (Additional file 3).

### Comparison of included and excluded mothers

Maternal demographics were similar between mothers who were included in our final analysis and those excluded because of positive HIV status or other reasons. Mothers with HIV were significantly less likely to have received SP during their pregnancy or delivery (68% vs 93% and 100% for HIV-positive vs. other excluded and included, respectively; *p*-value < 0.001). Excluded mothers were also less likely to come to all four ANC visits (40% and 33% vs 61% for HIV-positive and other excluded vs included, respectively; *p*-value < 0.001) (Additional file 4).

## Discussion

We assessed whether malaria exposure *in utero* reduced the transplacental transfer of KSHV antibodies in a Kenyan cohort of mother-child pairs. Our results suggest there was no significant interference of malaria infection during pregnancy on the transfer of KSHV antibodies from mother to neonate nor any interaction between malaria infection during pregnancy and overall KSHV antibody levels in pregnant women at delivery.

Previous studies have found that malaria impedes the transfer of antibodies against *S. pneumonia*, measles [7, 8], tetanus [4, 6], HSV-1, VZV, and RSV [5], though contradictory results do exist [7, 8]. We found no significant difference in KSHV antibody transfer dependent on malaria during pregnancy, no matter how malaria was defined. Our findings could be the result of unidentified intrinsic or biological factors that led to adequate transplacental transfer of KSHV antibodies regardless of malaria infection. It may also be due to our definition of malaria during pregnancy. Most studies have focused on placental malaria and reduced transplacental antibody transfer. Associations with placental malaria suggest the timing of infection may be an influential factor affecting antibody transfer, though we had too few mothers with placental malaria (*n* = 4) to examine this relationship. We did look at the gestational age of the most recent infection prior to delivery and still reported no effect on transplacental KSHV antibody transfer.

KSHV antibodies may be resistant to barriers of transplacental transfer caused by malaria, such as placental inflammation, reduced Fc receptor binding avidity, or induced hypergammaglobulinemia which is known to reduce transplacental IgG transfer [15, 16]. Antibodies against KSHV may be better at competing for binding to the finite FcRn receptors and thus are easily transported across the placenta. It is also possible that the concentration of antibodies against KSHV are low enough that they do not saturate the FcRn receptors. The precise mechanisms of differential transfer of antibodies needs to be investigated further.

EBV is a gammaherpesvirus that is also endemic to sub-Saharan Africa. Up to 50% of children are infected with KSHV by two years of age [3] but almost all children have seroconverted for EBV in the same time period [17–19], denoting a longer delay in KSHV seroconversion in children from similar regions. Transplacental transfer of anti-VCA-p18 and anti-EBNA1 EBV antibodies as measured by multiplex-based suspension bead assay was found to be reduced by malaria infection during pregnancy in this same study population [9]. However, in this study, we focused on only the KSHV seropositive mothers and thus did not analyze the same mother-child pairs that were included in the EBV antibody analysis by Ogolla et al. [9], thus preventing a direct comparison between the two results. Nonetheless, the differences in effects of malaria on EBV versus KSHV antibodies transfer is striking and points to, as of yet, unidentified differences in the humoral response to these two viruses. The fact that we found no effect of malaria infection on KSHV antibody transfer from mother to child may be one of the reasons for the delayed seroconversion with KSHV in children in this same population. Thus, the data reported by this study may suggest that the infants received adequate levels of antibodies and that the maternal antibodies are adequately protecting infants from KSHV in early infancy.

Women with malaria infection during pregnancy were significantly more likely to come for all four ANC visits versus their uninfected counterparts. Women who came for fewer ANC visits may have been miscategorized into the never malaria group due to less opportunities to detect the parasite. If *in utero* malaria reduces KSHV antibody transplacental transfer, then our estimates may be driven towards the null due to differential misclassification, though we believe this misclassification occurred minimally, if at all. Symptomatic women would have received appropriate diagnosis and care for malaria infection at the study clinic. In addition, our use of qPCR, which allowed us to detect both asymptomatic and symptomatic malaria infections, minimized any potential misclassification. We found a high MIR among women in our study of 4.7 per 100 person-weeks. Chulaimbo District, where our study took place, has one of the highest MIRs in Kenya [20]. In addition, our use of qPCR to detect malaria episodes made our study more sensitive to malaria infections which may also account for the high incidence rate.

The longitudinal design of this study allowed us to look throughout pregnancy for *in utero* malaria infection and not just at malaria during delivery. By using both the multiplex and ELISA assays to measure antibody levels, we were able to examine a wider breadth of antibodies to KSHV antigens. The fact that we found similar results using both types of assays strengthens our conclusion that malaria does not appear to affect KSHV transplacental antibody transfer.

We had a limited sample size to adjust for multiple comparisons. Even so, our estimates were quite insignificant, and it is likely that if our sample size was larger, we would only have detected small associations, if any, between malaria infection during pregnancy and KSHV antibody transfer. We were unable to differentiate the exact number of malaria infections detected using qPCR and so created an algorithm to calculate a malaria incidence rate. Our sample only included women who delivered at the study hospital, who may be different from those lost to follow-up and were not generalizable to the rest of the population. Nonetheless, our sensitivity analysis comparing women included in our analysis to those excluded showed no differences in demographics or health characteristics.

## Conclusions

Malaria has been previously associated with KSHV infection and non-bed net use with KS disease in sub-Saharan Africa [2, 21–26]. We hypothesized that one of the mechanisms driving the association between malaria and KSHV infection in sub-Saharan Africa was through *in utero* malaria exposure that altered transplacental transfer of maternal IgG; the neonates first line of defense against infection. However, the data from this study suggests that malaria infection during pregnancy does not affect maternal KSHV antibody transfer to infants. Further studies should explore other potential biological mechanisms between malaria and KSHV including malaria’s potential role to increase susceptibility to and/or reactivation of KSHV during infancy and its potential role in the etiology of KS disease.

## Supplementary Information

**Additional file 1.** Inclusion criteria for analysis of malaria during pregnancy and transplacental KSHV antibody transfer.

**Additional file 2.** Relationship between maternal Kaposi sarcoma-associated herpesvirus (KSHV) antibody levels at delivery and malaria infection during pregnancy.

**Additional file 3.** Linear regression estimates of mean difference in KSHV antibody log(CMR) by malaria exposure.

**Additional file 4.** Maternal demographics, clinical characteristics, and pregnancy history by inclusion and exclusion status.

## Abbreviations

ANC: Antenatal clinic
CHAP study: Chulaimbo Antenatal Postnatal study
COMIRB: Colorado Multiple Institutional Review Board
CMR: Cord to maternal blood ratio
ELISA: Enzyme-linked immunosorbent assays
EBV: Epstein-Barr virus
FDR adjustment: False discovery rate adjustment
HIV: Human immunodeficiency virus
HSV-1: Herpes simplex virus 1
IgG: Immunoglobulin G
IVB: Intervillous blood
KS: Kaposi sarcoma
KSHV: Kaposi sarcoma-associated herpesvirus
KEMRI: Kenya Medical Research Institute
MIR: Malaria incidence rate
MFI: Median fluorescence intensity
ORF: Open reading frame
ODs: Optical densities
PBMCs: Peripheral blood mononuclear cells
PBS: Phosphate buffered saline
*Pf*: *Plasmodium falciparum*
qPCR: Quantitative polymerase chain reaction
RSV: Respiratory syncytial virus
SUNY: State University of New York
SP: Sulfadoxine-pyrimethamine
USA: United States of America
VZV: Varicella-zoster virus
