# Supplementary figures and images for: Malaria during pregnancy and transplacental transfer of Kaposi sarcoma-associated herpesvirus (KSHV) antibodies: a cohort study of Kenyan mother and child pairs
Source: Infect Agent Cancer. 2020 Nov 26;15:71. doi: 10.1186/s13027-020-00336-1 (PMC7690029; doi:10.1186/s13027-020-00336-1)

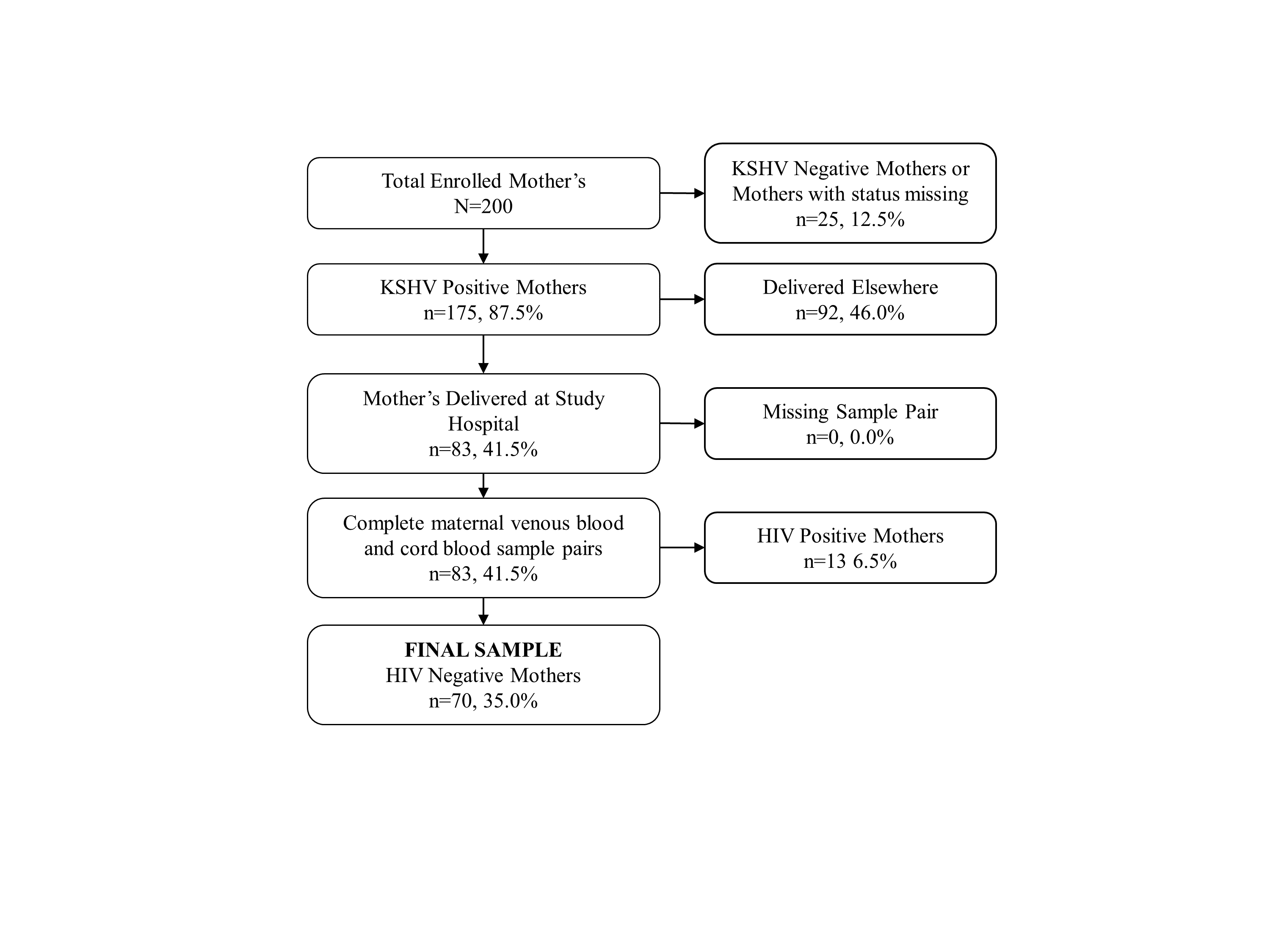

Supplement: Supplementary file 1 — Additional file 1. Inclusion criteria for analysis of malaria during pregnancy and transplacental KSHV antibody transfer. [file 13027_2020_336_MOESM1_ESM.png]
