# Supplementary material for: Malaria during pregnancy and transplacental transfer of Kaposi sarcoma-associated herpesvirus (KSHV) antibodies: a cohort study of Kenyan mother and child pairs
Source: Infect Agent Cancer. 2020 Nov 26;15:71. doi: 10.1186/s13027-020-00336-1 (PMC7690029; doi:10.1186/s13027-020-00336-1)
Supplement: Supplementary file 2 — Additional file 2. Relationship between maternal Kaposi sarcoma-associated herpesvirus (KSHV) antibody levels at delivery and malaria infection during pregnancy. [file 13027_2020_336_MOESM2_ESM.docx]

| **Additional File 2:** Relationship between maternal Kaposi sarcoma-associated herpesvirus (KSHV) antibody levels at delivery and malaria during pregnancy. | | | | | | | | | | | |
| --- | --- | --- | --- | --- | --- | --- | --- | --- | --- | --- | --- |
|  | **Mann Whitney U test of any vs. no malaria during pregnancy or delivery and maternal KSHV antibody levels (n=70)** | | | | **Mann Whitney U test of time of most recent malaria exposure prior to delivery and maternal KSHV antibody levels (n=38)** | | | | **Spearman's correlation of MIR^a^ and maternal KSHV antibody levels (n=38)** | | |
| **Antibody specificity** | **No Malaria (n=32)** | **Any Malaria (n=38)** |  |  | **Exposure ≤26 weeks (n=14)** | **Exposure >27 weeks (n=24)** |  |  |  |  |  |
| **ELISA** | **Median (IQR)** | **Median (IQR)** | **p-value** | **p-value (FDR)** | **Median (IQR)** | **Median (IQR)** | **p-value** | **p-value (FDR)** | **Rho (ρ)** | **p-value** | **p-value (FDR)** |
| K8.1 | 2.22(1.24,2.70) | 2.57(0.72,3.33) | 0.49 | 0.64 | 2.05(0.42,3.27) | 2.62(0.81,3.35) | 0.58 | 0.67 | 0.19 | 0.26 | 0.46 |
| ORF73 | 0.91(0.45,1.97) | 1.93(1.05,2.42) | 0.01* | 0.09 | 1.89(0.56,2.17) | 1.93(1.21,3.07) | 0.14 | 0.26 | 0.14 | 0.42 | 0.49 |
| **Multiplex** | **Median (IQR)** | **Median (IQR)** | **p-value** | **p-value**  **(FDR)** | **Median (IQR)** | **Median (IQR)** | **p-value** | **p-value (FDR)** | **Rho (ρ)** | **p-value** | **p-value (FDR)** |
| K8.1 | 928(541,1546) | 1290(255,4760) | 0.43 | 0.64 | 779(110,4521) | 2402(333,5496) | 0.15 | 0.26 | 0.33 | 0.04* | 0.28 |
| ORF73 | 1608(570,3707) | 3242(1447,4539) | 0.05* | 0.15 | 2441(657,3492) | 3622(1547,4962) | 0.08 | 0.26 | 0.19 | 0.26 | 0.46 |
| K10.5 | 701(352,2175) | 618(319,3843) | 0.64 | 0.64 | 600(291,3635) | 618(366,4822) | 0.50 | 0.67 | -0.01 | 0.99 | 0.99 |
| ORF38 | 198(109,434) | 214(140,349) | 0.58 | 0.64 | 209(118,444) | 214(142,329) | 0.73 | 0.73 | 0.29 | 0.08 | 0.28 |
| ORF50 | 1452(552,3406) | 2653(960,4025) | 0.06 | 0.15 | 2248(584,3222) | 3047(1224,4204) | 0.11 | 0.26 | 0.15 | 0.39 | 0.49 |
| Association estimates between any malaria exposure during pregnancy or delivery (n=70), and among mothers with malaria exposure (n=38), the relationship between time of most recent malaria exposure (**≤**26 weeks gestation (n=14) vs. >27 weeks gestation (n=24)) or malaria incident rate (MIR)(n=38) and maternal venous blood Kaposi sarcoma-associated herpesvirus (KSHV) antibody levels at delivery among pregnant women enrolled in the Chulaimbo Antenatal Postnatal (CHAP) study, Kenya, 2011  *p-values<0.05 considered statistically significant. ^a^MIR calculated as the number of total maternal malaria episodes during pregnancy per 100 person-weeks donated to the study.  Abbreviations Used: enzyme-linked immunosorbent assays (ELISA), false discovery rate (FDR), interquartile range (IQR), Kaposi sarcoma-associated herpesvirus (KSHV), malaria incidence rate (MIR), open reading frame (ORF). | | | | | | | | | | | |
