# Supplementary material for: Malaria during pregnancy and transplacental transfer of Kaposi sarcoma-associated herpesvirus (KSHV) antibodies: a cohort study of Kenyan mother and child pairs
Source: Infect Agent Cancer. 2020 Nov 26;15:71. doi: 10.1186/s13027-020-00336-1 (PMC7690029; doi:10.1186/s13027-020-00336-1)
Supplement: Supplementary file 3 — Additional file 3. Linear regression estimates of mean difference in KSHV antibody log(CMR) by malaria exposure. [file 13027_2020_336_MOESM3_ESM.docx]

| **Additional File 3:** Linear regression estimates of mean difference in KSHV antibody log(CMR) by malaria exposure | | | | | | | | | | | | | |
| --- | --- | --- | --- | --- | --- | --- | --- | --- | --- | --- | --- | --- | --- |
|  | **Any vs. no malaria during pregnancy or delivery and CMR (n=70)** | | | **Time of most recent malaria exposure prior to delivery and CMR (n=38)** | | | | | | **MIR^1^ and CMR (n=38)** | | |  |
|  | **Crude (no adjustment required)** | | | **Crude** | | | **Adjusted^2^** | | | **Crude (no adjustment required)** | | |  |
| **ELISA** | **Mean Diff (sd)** | **p-value** | **p-value (FDR)** | **Mean Diff (sd)** | **p-value** | **p-value (FDR)** | **Mean Diff (sd)** | **p-value** | **p-value (FDR)** | **Mean Diff (sd)** | **p-value** | **p-value (FDR)** |  |
| K8.1 | 0.05(0.11) | 0.66 | 0.97 | -0.27(0.18) | 0.15 | 0.51 | -0.17(0.22) | 0.43 | 0.86 | 0.31(0.18) | 0.10 | 0.35 |  |
| ORF73 | 0.01(0.13) | 0.97 | 0.97 | -0.23(0.19) | 0.22 | 0.51 | -0.11(0.22) | 0.62 | 0.86 | 0.09(0.19) | 0.66 | 0.77 |  |
| **Multiplex** | **Mean Diff (sd)** | **p-value** | **p-value (FDR)** | **Mean Diff (sd)** | **p-value** | **p-value (FDR)** | **Mean Diff (sd)** | **p-value** | **p-value (FDR)** | **Mean Diff (sd)** | **p-value** | **p-value (FDR)** |  |
| K8.1 | -0.01(0.10) | 0.97 | 0.97 | -0.22(0.14) | 0.14 | 0.51 | -0.20(0.17) | 0.27 | 0.86 | 0.11(0.15) | 0.46 | 0.64 |  |
| ORF73 | -0.02(0.11) | 0.83 | 0.97 | -0.03(0.18) | 0.87 | 0.87 | -0.04(0.22) | 0.86 | 0.86 | -0.14(0.18) | 0.45 | 0.64 |  |
| K10.5 | -0.17(0.14) | 0.25 | 0.88 | 0.20(0.25) | 0.43 | 0.75 | 0.29(0.30) | 0.34 | 0.86 | -0.54(0.24) | 0.03* | 0.23 |  |
| ORF38 | -0.14(0.11) | 0.19 | 0.88 | -0.09(0.17) | 0.60 | 0.83 | 0.07(0.20) | 0.75 | 0.86 | -0.20(0.17) | 0.26 | 0.61 |  |
| ORF50 | -0.06(0.11) | 0.61 | 0.97 | -0.04(0.17) | 0.83 | 0.87 | -0.09(0.20) | 0.66 | 0.86 | -0.01(0.17) | 0.98 | 0.98 |  |
| Crude and adjusted linear regression estimates of the mean difference in log transformed cord to maternal blood ratio (CMR) of KSHV antibody levels for women with any malaria (n=38) vs. no malaria (n=32), and among women with malaria during pregnancy or delivery, the average difference in log(CMR) of KSHV antibody levels comparing time of most recent malaria exposure (27+ weeks gestation (n=24) vs. <27 weeks gestation (n=14)) and malaria incidence rate (MIR) per 100 person-weeks (n=38) among mother-child pairs enrolled in the Chulaimbo Antenatal Postnatal (CHAP) study, Kenya, 2011.  All CMR data were log transformed.  *p-values<0.05 considered statistically significant. ^1^MIR calculated as the number of total maternal malaria episodes during pregnancy per 100 person-weeks donated to the study.  ^2^Adjusted for whether child was preterm (<38 weeks gestational age vs 38+ weeks gestational age).  Abbreviations Used: cord to maternal blood ratio (CMR), enzyme-linked immunosorbent assays (ELISA), false discovery rate (FDR), Kaposi sarcoma-associated herpesvirus (KSHV), malaria incidence rate (MIR), mean difference (mean diff), open reading frame (ORF), standard deviation (sd). | | | | | | | | | | | | | |
