# Supplementary material for: Malaria during pregnancy and transplacental transfer of Kaposi sarcoma-associated herpesvirus (KSHV) antibodies: a cohort study of Kenyan mother and child pairs
Source: Infect Agent Cancer. 2020 Nov 26;15:71. doi: 10.1186/s13027-020-00336-1 (PMC7690029; doi:10.1186/s13027-020-00336-1)
Supplement: Supplementary file 4 — Additional file 4. Maternal demographics, clinical characteristics, and pregnancy history by inclusion and exclusion status. [file 13027_2020_336_MOESM4_ESM.docx]

| **Additional File 4:** Maternal demographics, clinical characteristics, and pregnancy history by inclusion and exclusion status (N=200) | | | | |  |  |
| --- | --- | --- | --- | --- | --- | --- |
|  | **Included (n=70)** | **Excluded HIV-Positive (n=25)** | **Excluded Other (n=105)** | **p-value** | | |
| **Mother's age at enrollment (mean) [sd]** | 22.0[6.2] | 24.7[5.4] | 22.3[5.2] | 0.12 | | |
| **Mother's tribe** |  |  |  |  | | |
| Luo | 61(87.1) | 22(88.0) | 83(79.0) | 0.61 | | |
| Luhya | 9(12.9) | 3(12.0) | 17(16.2) |  | | |
| Other | 0(0.0) | 0(0.0) | 5(4.8) |  | | |
| **Maternal education:** Upper primary school or higher vs. Lower primary school or lower | 55(78.6) | 17(68.0) | 84(80.0) | 0.42 | | |
| **Marital status:** Married vs Single/widowed | 47(67.1) | 18(72.0) | 67(63.8) | 0.80 | | |
| **Maternal bed net use:** Yes vs. No | 68(97.1) | 25(100.0) | 101(96.2) | 1.00 | | |
| **Maternal gravidity** |  |  |  |  | | |
| Nulliparous | 25(35.7) | 7(28.0) | 43(41.0) | 0.69 | | |
| Primiparous | 22(31.4) | 9(36.0) | 26(24.8) |  | | |
| Multiparous | 23(32.9) | 9(36.0) | 35(33.3) |  | | |
| **Worm infection during pregnancy** |  |  |  |  | | |
| Yes | 16(22.9) | 4(16.0) | 12(11.4) | 0.16 | | |
| No | 47(67.1) | 18(72.0) | 78(74.3) |  | | |
| Missing | 7(10.0) | 3(12.0) | 15(14.3) |  | | |
| **Hypergammaglobulinemia (Adjusted venous blood concentration)** |  |  |  |  | | |
| > 30mg/ml | 19(27.1) | 5(20.0) | 4(3.8) | 0.65 | | |
| ≤ 30mg/ml | 37(52.9) | 5(20.0) | 8(7.6) |  | | |
| Missing | 14(20.0) | 15(60.0) | 93(88.6) |  | | |
| **Sulfadoxine-pyrimethamine (SP) given during pregnancy or delivery** | 70(100.0) | 17(68.0) | 98(93.3) | <0.001* | | |
| **Total antenatal clinic visits** |  |  |  |  | | |
| 1 | 4(5.7) | 7(28.0) | 20(19.0) | <0.001* | | |
| 2 | 6(8.6) | 2(8.0) | 26(24.8) |  | | |
| 3 | 17(24.3) | 6(24.0) | 24(22.9) |  | | |
| 4 | 43(61.4) | 10(40.0) | 35(33.3) |  | | |
| **Any malaria during pregnancy** | 38(54.3) | 12(48.0) | 56(53.3) | 0.86 | | |
| Comparison of maternal demographics, clinical characteristics, and pregnancy history by inclusion or exclusion in the final analytic sample, Chulaimbo Antenatal Postnatal (CHAP) study, Kenya, 2011 (N=200)  Data are presented as number (percent) unless otherwise specified.  *p-values <0.05 considered statistically significant.  **All variables have complete data except for marital status which is missing for 2(1.9%) women excluded for reasons other than being HIV-positive and maternal gravidity which is missing for 1(1.0%) woman excluded for reasons other than being HIV-positive.  Abbreviations Used: human immunodeficiency virus (HIV). | | | | | |  |
